# The role of prospero homeobox 1 (PROX1) expression in follicular thyroid carcinoma cells

**DOI:** 10.18632/oncotarget.23167

**Published:** 2017-12-12

**Authors:** Magdalena Rudzinska, Joanna K. Ledwon, Damian Gawel, Justyna Sikorska, Barbara Czarnocka

**Affiliations:** ^1^ Department of Biochemistry and Molecular Biology, Centre of Postgraduate Medical Education, Warsaw, Poland

**Keywords:** PROX1, thyroid cancer, follicular thyroid carcinoma, invasion, cytoskeleton

## Abstract

The prospero homeobox 1 (Prox1) transcription factor is a key player during embryogenesis and lymphangiogenesis. Altered Prox1 expression has been found in a variety of human cancers, including papillary thyroid carcinoma (PTC). Interestingly, Prox1 may exert tumor suppressive or tumor promoting effect, depending on the tissue context. In this study, we have analyzed Prox1 expression in normal and malignant human thyroid carcinoma cell lines. Moreover, we determined the effect of Prox1 silencing and overexpression on the cellular processes associated with the metastatic potential of tumor cells: proliferation, migration, invasion, apoptosis and anchorage-independent growth, in the follicular thyroid carcinoma (FTC) FTC-133 cell line. We found that Prox1 expression was significantly higher in FTC-derived cells than in PTC-derived cells and normal thyroid, and it was associated with the PI3K/Akt signaling pathway. In the FTC-133 cells, it was associated with cell invasive potential, motility and wound closure capacities, but not with proliferation or apoptosis. Modifying Prox1 expression also induced substantial changes in the cytoskeleton structure and cell morphology. In conclusion, we have shown that Prox1 plays an important role in the development of FTC and that its suppression prevents, whereas its overexpression promotes, the malignant behavior of thyroid follicular cancer cells.

## INTRODUCTION

Differentiated thyroid carcinoma (DTC) originates from thyroid follicular cells and is the most common endocrine malignancy. Two major DTC histopathological types: papillary (PTC) and follicular thyroid carcinoma (FTC), account for up to 90% of all thyroid tumor cases. PTC has a tendency to metastasize to regional lymph nodes and disseminate through lymphatic vasculature, whereas FTC rather via the circulatory system. However, the mechanisms involved in the lymphatic dissemination of cancer cells are poorly understood.

Identifying molecular markers of the lymphatic endothelial cells and lymphatic signaling factors has facilitated studies of their impact on the tumor growth and metastasis. Well-known markers of lymphatic cells include the mucine-type transmembrane glycoprotein podoplanin, hyaluronian receptor LYVE-1, and vascular endothelial growth factor receptor 3 (VEGFR-3) as well as the prospero homeobox 1 (Prox1) transcription factor [1-4].

*PROX1* (1q32.2-32.3) belongs to a homeodomain family of transcription factors. It is a mammalian homolog of the *Drosophila* prospero gene which regulates the nuclear localization of Prospero and acts as a tumor suppressor by preventing neuroblast self-renewal [5, 6]. The Prox1 protein plays an essential role in embryogenesis and in the development of various organs and tissues [7, 8]. Its expression was found in normal tissues, such as lens, heart, liver, kidney, skeletal muscles, pancreas, and central nervous system, at different developmental stages [9-16]. *PROX1* is also known as a master control gene for lymphangiogenesis during early embryonic development [17]. Prox1 is not only a marker of lymphatic endothelial cells (LEC) but it also plays a pivotal role in determining the lymphatic endothelial cells characteristics and their destiny [4, 17]. It has been reported that Prox1 may function either as an activator of gene transcription by direct binding of its homeodomain to specific DNA elements, or as a co-repressor [18-23].

In a variety of malignancies, tumor progression is associated with changes in cell adhesion, activation of epithelial–mesenchymal transition, and with various biochemical alterations. These modifications have an effect on the biological properties of the cells, their behavior and the changes associated with the cancer cell phenotype, such as enhanced migratory capacity, invasiveness or elevated resistance to apoptosis. Results of several studies indicate that Prox1 is implicated in controlling at least some of essential cellular processes, such as cell differentiation, proliferation, migration, and apoptosis. Moreover, recent studies have suggested that Prox1 may also play a role in tumor development and progression as altered *PROX1* expression (on both transcript and protein level) has been found in a variety of human cancers, such as brain tumors, pancreatic cancer, colon cancer, liver carcinoma, Kaposi sarcoma and small cell lung carcinoma [24-31]. However, its exact role in carcinogenesis is yet unclear with some researchers reporting its possible tumor-promoting role and some others suggesting its tumor suppressive function [24, 25, 28, 30, 32-38]. This suggests that Prox1 may function either as a suppressor gene, or as an oncogene, depending on the tissue and cancer type context. In PTC, *PROX1* has been shown to be inactivated through mRNA downregulation and cytoplasmic mislocalization, and this inactivation substantially promoted the malignant behavior of the tumor [39]. However, up to date there have been no studies on the expression of the *PROX1* gene and the role of its protein product in the follicular thyroid tumors.

In this study, we have analyzed the expression of Prox1 in normal and malignant human thyroid cells. Moreover, in order to determine whether the *PROX1* gene is involved in thyroid cancer progression, we determined the effect of *PROX1* silencing and overexpression on the cellular processes associated with the metastatic potential of tumor cells, such as proliferation, migration, invasion, apoptosis and anchorage-independent growth, in the FTC-133 human follicular thyroid carcinoma cell line.

## RESULTS

### *PROX1* expression

We analyzed the expression levels and distribution of Prox1 in four thyroid cancer cell lines: TPC1 and BcPAP derived from papillary thyroid carcinoma, and FTC-133 and CGTH-W-1 derived from follicular thyroid carcinoma, as well as in the normal thyroid NTHY cell line, using quantitative real-time reverse transcription-PCR (Q-RT-PCR), Western blot and immunofluorescent analyses. The HepG2 cells which express high levels of the Prox1 protein were used as a positive control.

**Figure 1:**
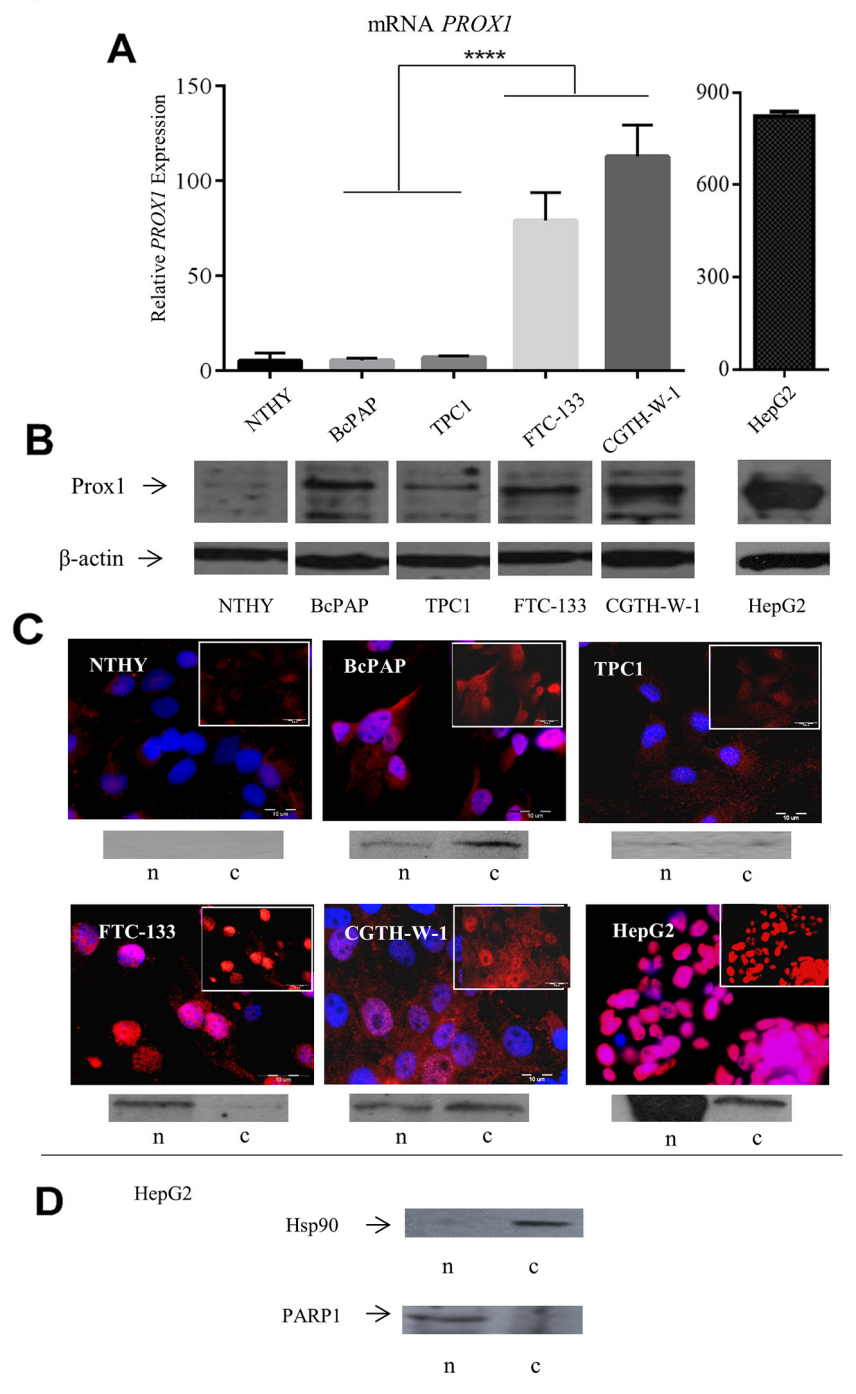
Expression of *PROX1* in thyroid carcinoma cell lines NTHY: normal thyroid, BcPAP and TPC1: papillary thyroid carcinoma-derived cell lines, FTC-133 and CGTH-W-1: follicular thyroid carcinoma-derived cell lines. **(A)** Relative *PROX1* mRNA expression in all cell lines. *PROX1* and β*-actin* mRNA levels were quantified and *PROX1* expression normalized against the expression of the *ACTB* housekeeping gene. Each bar represents the mean of triplicate measurements on three different samples for each cell line. Statistical significance was evaluated by paired Student’s t-test using the GraphPad Prism software. The corresponding *P*-value (^****^: *P*-value < 0.0001) is presented. **(B)** The Prox1 protein levels in the tested cell lines by Western blot. Total protein extracts from logarithmically growing cells were analyzed. ß-actin was used as a loading control. **(C)** Immunofluorescent labeling of the Prox1 protein (red) and its cellular localization in the analyzed cell lines. Inset: the Prox1 content and cellular localization after DAPI (blue) removal (scale bar: 10μm). All pictures were taken at a x 1000 magnification. Below: Prox1 protein content in nuclear (n) and cytoplasmic (c) fractions determined by Western blot. **(D)** Immunoblot analysis of Hsp90 (control for the cytoplasmic fraction) and PARP1 (control for nuclear fraction) protein expression in nuclear (n) and cytoplasmic (c) fractions isolated from the HepG2 cells.

The *PROX1* gene expression varied between the studied cell lines, with the highest transcript levels in the CGTH cell line (26 times higher than in the normal thyroid NTHY cells), followed by the FTC-133 cells (16 times higher). The mRNA levels in these two follicular carcinoma cell lines were significantly higher than in the two papillary carcinoma cell lines, TPC1 and BcAP (*P*-value < 0.0001), in which the transcript levels were comparable to those detected in the NTHY cells (Figure 1A).

The Prox1 protein expression was observed in all the five cell lines. In the two follicular thyroid carcinoma cell lines: FTC-133 and CGTH, the protein levels detected by Western blot paralleled the results of Q-RT-PCR, with strong bands corresponding to the protein detected in both cell lines. In contrast, in TPC1 and BcPAP cells, the Prox1 protein levels did not correlate with the mRNA transcript levels: the protein bands of different intensities were detected by Western blot irrespectively of the mRNA quantity. A faint band corresponding to the Prox1 protein was also observed in the NTHY cells. In the same line, the immunoblotting results confirmed those of Q-RT-PCR for the two follicular-type cell lines (Figure 1B).

The cellular distribution of the Prox1 protein was then determined by immunofluorescent staining, and Western blot of nuclear and cytoplasmic cell fractions. The Prox1 protein cellular localization varied between the studied cell lines. In TPC1 cells, the Prox1 expression was observed only in the cytoplasm, with a weak and diffuse immunofluorescent signal. In contrast, in BcPAP cells the Prox1 staining in the cytoplasm was stronger and an intensive nuclear signal was observed. A strong nuclear staining was also detected in both follicular carcinoma-derived cell lines: FTC-133 and CGTH. However, the CGTH cells additionally presented a strong staining in the cytoplasm, with a nearly even signal distribution between the two compartments, whereas in the FTC-133 cells the cytoplasmic staining was weaker or even negligible compared to the nuclear staining. The cellular distribution of the Prox1 expression was similar between the CGTH and BcPAP cells, however the overall protein levels in the latter cell line were much lower. In the normal thyrocytes (NTHY cell line), feeble cytoplasmic Prox1 staining was detected. Such a distribution of Prox1 expression in the studied cell lines was confirmed by Western blot analysis of nuclear and cytoplasmic fractions isolated from whole cell lysates. The presence of Prox1 protein bands and their intensity corresponded to the cell compartment localization and the intensity of Prox1 labeling detected by immunofluorescent staining (Figure 1C).

### Prox1 function

In order to determine the biological significance of Prox1 expression for malignant thyroid cancer cells, we analyzed possible interactions of the Prox1 expression with the PI3K/Akt signaling pathway. We also studied migratory and invasive behavior, anchorage-independent growth, viability, and apoptosis of the FTC-133 cells in which *PROX1* had been either silenced (“loss-of-function” study) or overexpressed (“gain-of function” study).

**Figure 2:**
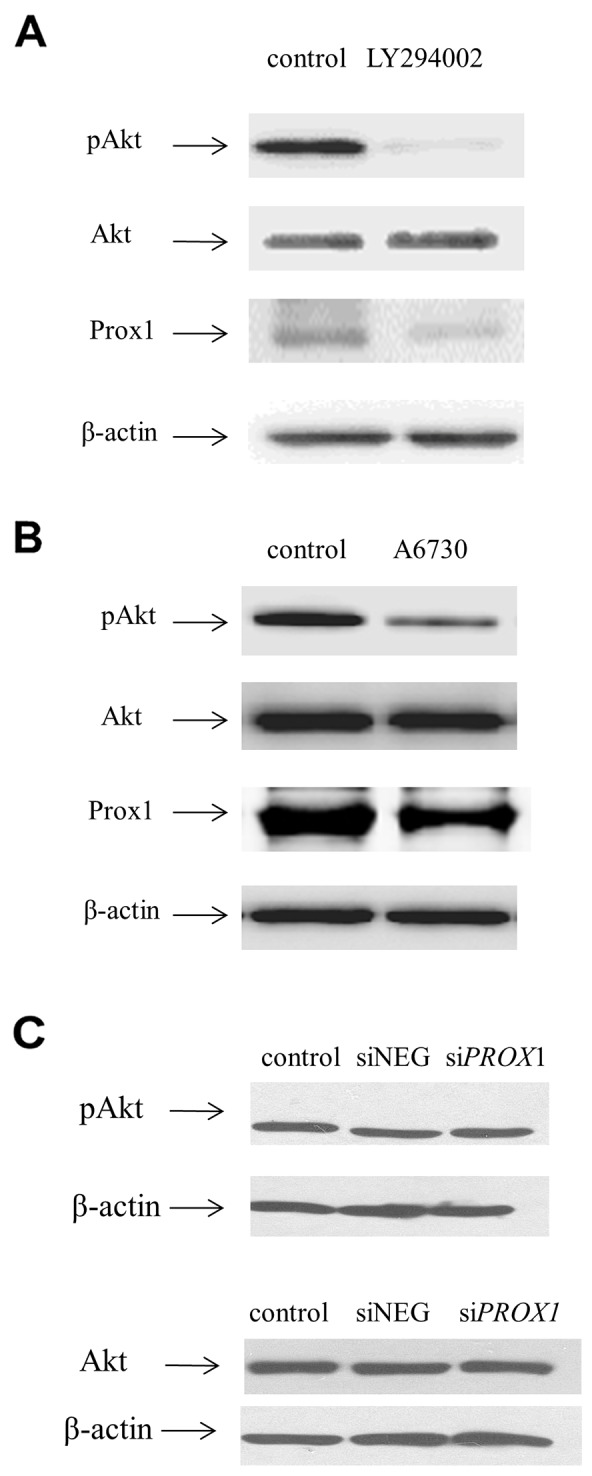
Inhibiting the PI3K/AKT pathway inhibits *PROX1* expression, as shown by Western blot FTC-133 cells were treated with LY204002 **(A)**, the PI3K inhibitor (50μM), or with A6730 **(B)**, the AKT1/AKT2 inhibitor (10 μM), for 48 hours. Negative control cells were grown with DMSO instead of the inhibitors. **(C)** AKT and phospho-AKT expression in FTC-133 cells after silencing of *PROX1*. The whole cell protein extracts were prepared and analyzed by Western blotting using a primary antibody against phospho-AKT (Ser473), AKT, or Prox1, followed by an incubation with relevant goat anti-rabbit or goat-anti mouse HRP-conjugated affinity-purified secondary antibodies. β-actin was used as a loading control.

We first analyzed whether there is a relationship between Prox1 expression and the PI3K/Akt signaling cascade which is involved in regulating cell growth, differentiation and oncogenic transformation, and is known to be constitutively active in FTC-133 cells due to the deficiency of PTEN. To this effect, we first checked the expression levels of AKT and phospho-AKT in the FTC-133 cells transfected with siNEG and si*PROX1.* The levels of both AKT and pAKT were not changed (Figure 2C). Then, we sequentially inhibited two elements of this pathway: PI3K with the LY294002 inhibitor and Akt1/Akt2 with A6730. Since the phosphorylation of AKT occurs downstream of PI3K activation, we first determined the levels of phospho-AKT (pAKT) in the FTC-133 cells cultured with either of the two inhibitors. We found that pAKT levels significantly decreased in cells cultured with the PI3K inhibitor (LY294002), whereas inhibition of AKT1/AKT2 by A6730, the Akt1/Akt2 inhibitor, reduced the pAKT levels less efficiently. These changes were consistent with changes in the Prox1 expression levels (Figure 2A-2B).

**Figure 3:**
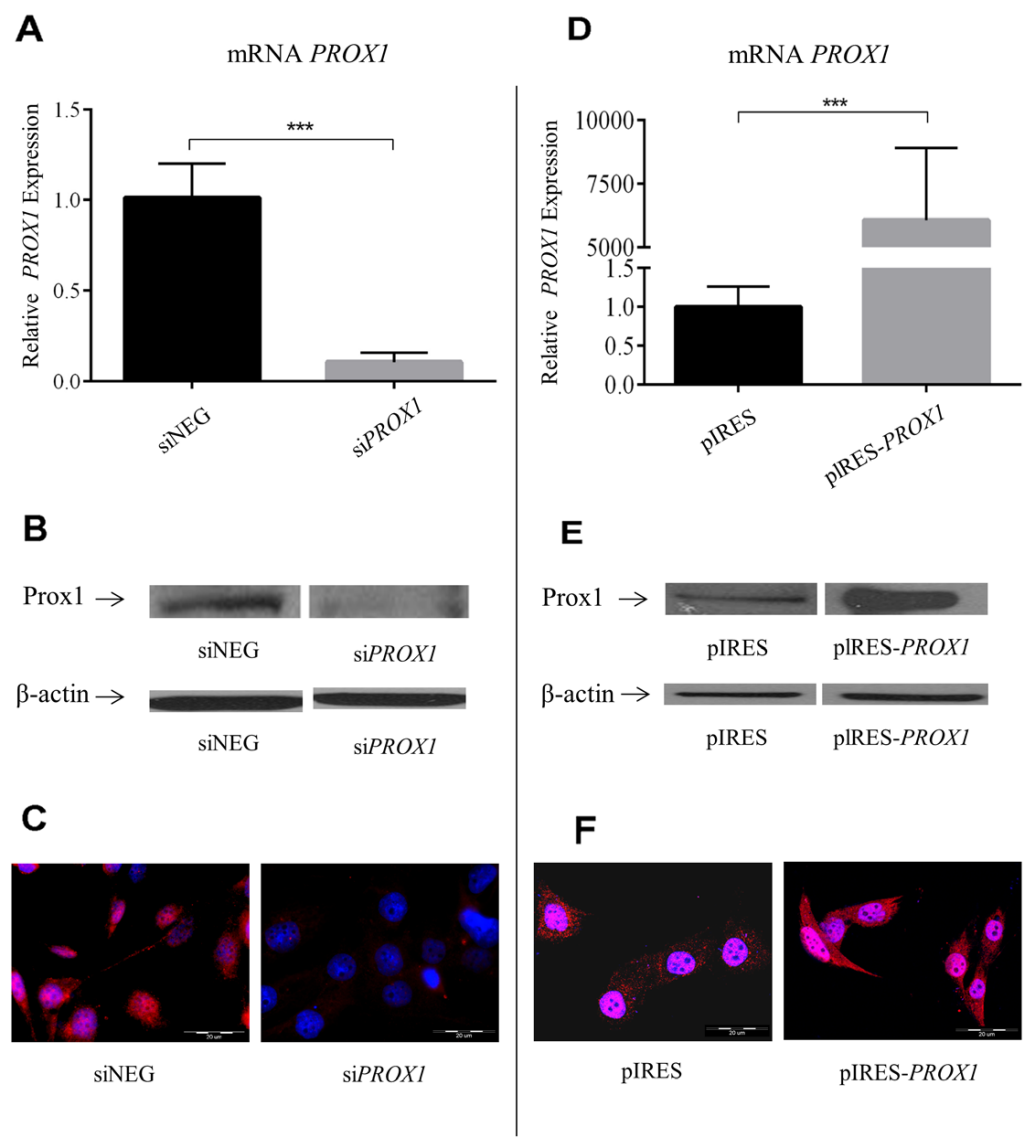
*PROX1* expression in FTC-133 cells following *PROX1* silencing with *PROX1*-specific siRNA **(A–C) or** *PROX1* overexpression **(D–F).** siNEG: negative control (cells transfected with negative siRNA). (A) and (D) *PROX1* mRNA levels determined by real-time quantitative PCR (RT-qPCR). The results were normalized against *ACTB* transcript levels and bars represent the average *PROX1* transcript levels in cells transfected with *PROX1*-siRNA compared to control cells. The results represent means from five independent experiments. (B) and (E) Prox1 protein levels determined by Western blotting, and (C and F) by immunofluorescent staining. Prox1 was detected with a rabbit anti-Prox1 polyclonal antibody and the DyLight 594-conjugated purified rabbit F(ab)_2_ anti-goat secondary antibodies (red), and the nuclei were stained with DAPI (blue).

Then, we studied the effect of *PROX1* silencing and overexpression on the phenotype and properties of the FTC-133 cells. We have silenced the expression of endogenous Prox1 in these cells using short interfering RNA (siRNA) against human *PROX1*. We initially tested two sets of siRNA, the one which gave a stronger silencing was selected for use in further experiments. As shown by real-time PCR analysis, the mean relative *PROX1* mRNA levels in cells treated with this *PROX1*-specific siRNA were reduced up to 10 times as compared to cells which were transfected with negative control siRNA with no known homology to mammalian genes (Figure 3A). The maximal silencing effect was observed 48 hours after transfection. These results were confirmed by Western blot analysis: the Prox1 protein was detected only in cells transfected with negative control siRNA (Figure 3B). In the same line, Prox1 immunoreactivity as detected by immunofluorescent staining was limited to the FTC-133 cells transfected with control negative siRNA (Figure 3C). In order to overexpress the *PROX1* gene in the FTC-133 cells, we transfected them with pIRES2-eYFP plasmid containing *PROX1*-cDNA, obtaining expression levels which were over six thousand higher than those detected in control cells transfected with an empty plasmid (FTC-133/pIRES cells; Figure 3D). These results were also confirmed by Western blot and immunofluorescence analyses (Figure 3E and 3F).

**Figure 4:**
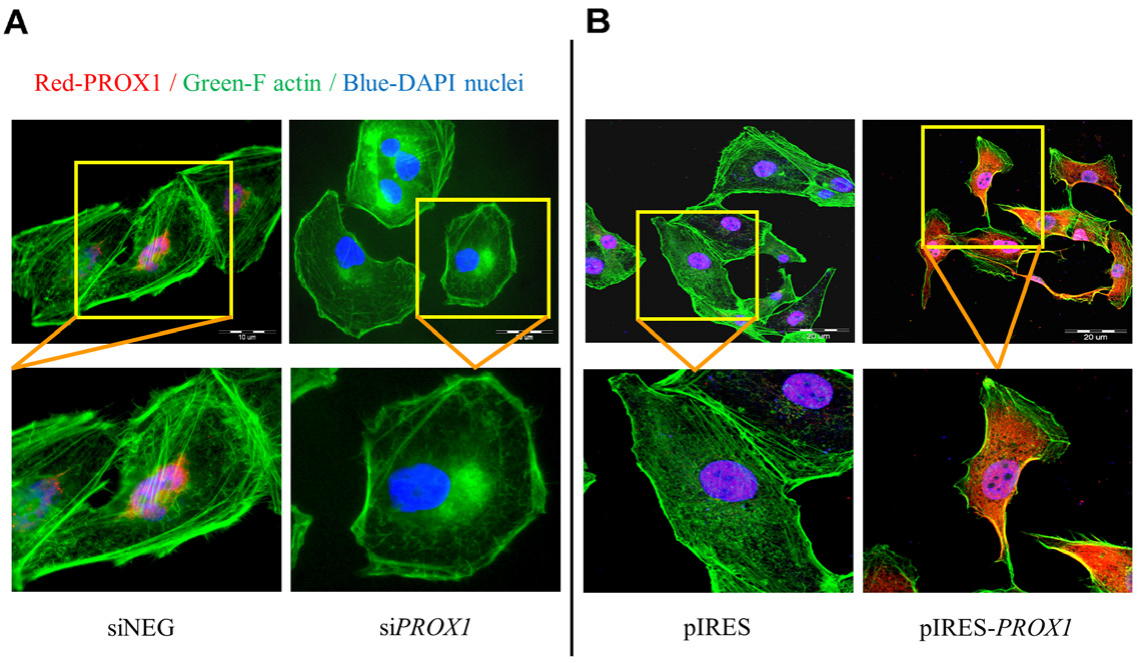
The effect of *PROX1* silencing **(A)** and overexpression **(B)** on the organization of actin cytoskeleton and cell morphology in FTC-133 cells. Following a transient transfection with si*PROX1*-RNA or pIRES-*PROX1*, the cells were stained with an anti-Prox1 antibody (red), with phalloidin (green) for F-actin and with DAPI (blue) for DNA. The yellow boxes depict areas which are zoomed in the bottom panel.

We found that *PROX1* silencing induced substantial changes in the cytoskeleton structure and cell morphology. The cells transfected with negative control siRNA showed more stress fibers compared to non-transfected cells but only minor morphological changes. In contrast, the FTC-133/si*PROX1* cells became rounder and flatter, amoeboid-like in shape, suggesting the epithelial cell phenotype. A majority of them have also undergone spatial modification of actin fibers, with the formation of specialized cell membrane structures, like filopodia and focal adhesion complexes. In addition, they have developed long cellular extensions (Figure 4A). Transient Prox1 overexpression in FTC-133 cells has also led to dramatic changes in the cell architecture and shape. In cells transfected with an empty plasmid, the actin stress filaments were redistributed on the peripheries, without significant changes in the cell shape. In contrast, the cells transfected with the pIRES-PROX1 construct have adopted a spindle-like mesenchymal-like form with peripheral polarized stress fibers redistributed around the cells and well visible actin caps on the endings of elongated protrusions (Figure 4B).

**Figure 5:**
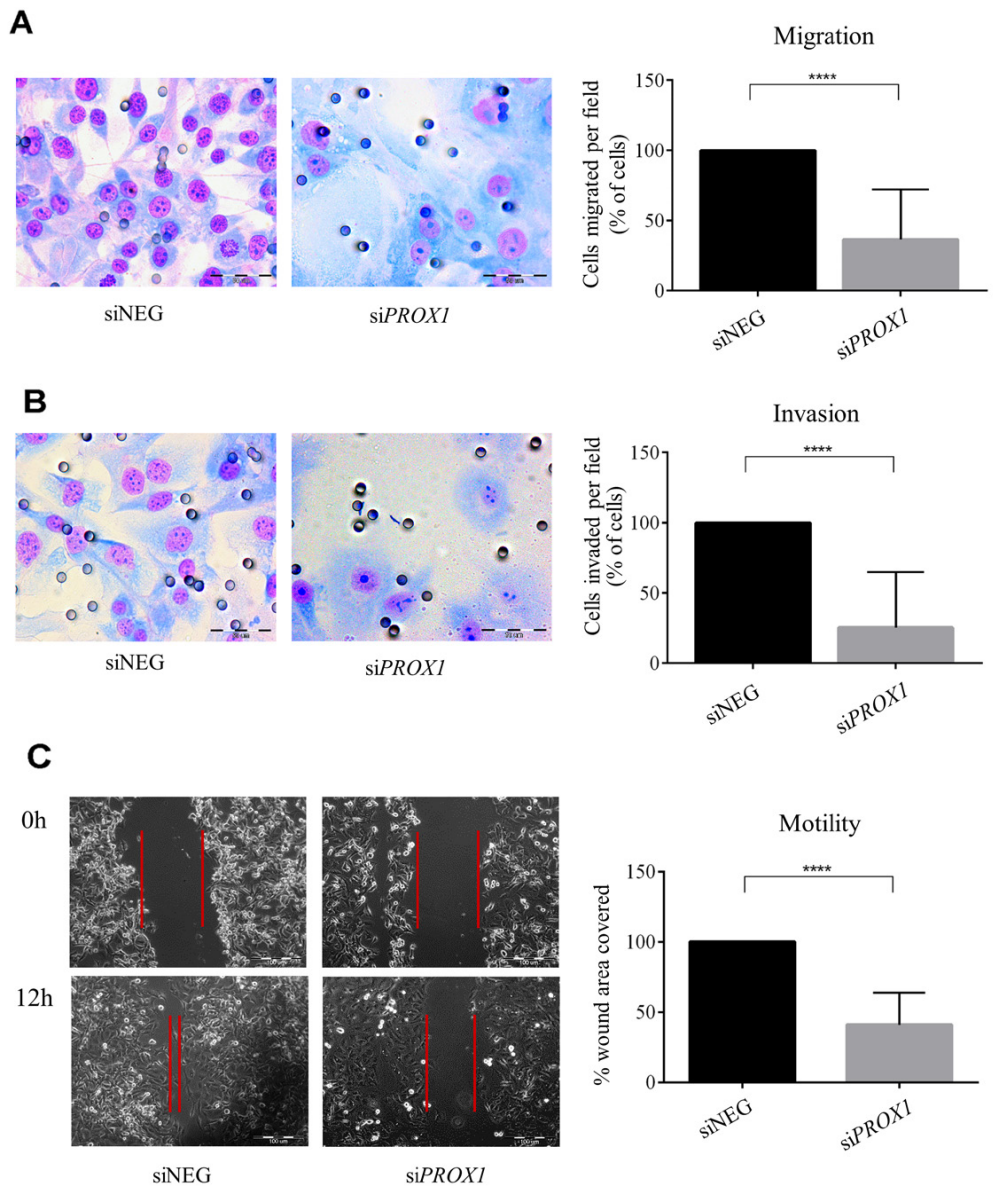
The effect of *PROX1* depletion on migration **(A)**, invasion **(B)** and motility **(C)** of FTC-133 cells, determined by chamber migration, Matrigel invasion, and wound-healing assay, respectively. Bar graphs illustrate the means ± standard deviation (SD) of the results from four independent experiments. Asterisks (^****^) denote the *P*-value < 0.0001. (A) and (B) To evaluate their migration and invasive potential, the FTC-133 cells transfected with si*PROX1* or control siNEG RNA were seeded in Boyden insert chambers or Matrigel Invasion Chambers with 8-μm pores. Lower chambers were filled with culture medium supplemented with 10% FBS. After 24 hours, the cells which have passed through membranes were fixed, stained, and photographed at a 40x magnification. (C) The wound closure percentage 24 hours after the scratch in monolayers of the FTC-133 cells transfected with si*PROX1* or control siNEG. Representative light microscope images were taken at a 200x magnification.

**Figure 6:**
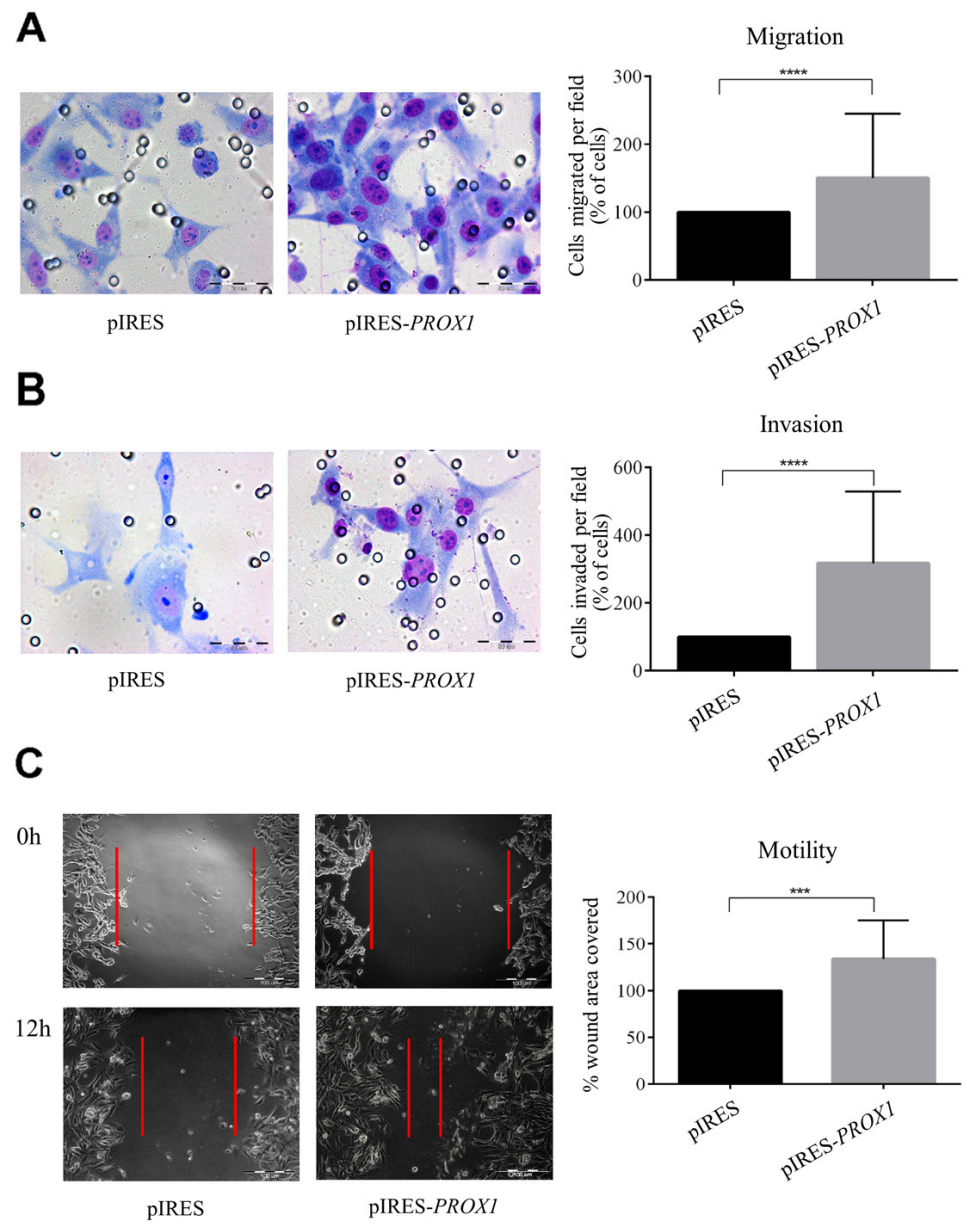
The effect of *PROX1* overexpression on migration **(A)**, invasion **(B)** and motility **(C)** of FTC-133 cells, determined by chamber migration, Matrigel invasion, and wound-healing assay, respectively. Bar graphs illustrate the means ± standard deviation (SD) of the results from four independent experiments. Asterisks denote the corresponding *P*-values (^****^: *P*-value < 0.0001; ^***^: *P*-value < 0.01) (A) and (B) To evaluate their migration and invasive potential, the FTC-133 cells transfected pIRES-*PROX1* plasmid or empty pIRES plasmid were seeded in Boyden insert chambers or Matrigel Invasion Chambers with 8-μm pores. Lower chambers were filled with culture medium supplemented with 10% FBS. After 24 hours, the cells which have passed through membranes were fixed, stained, and photographed at a 40x magnification. (C) The wound closure percentage 24 hours after the scratch in monolayers of the FTC-133 cells transfected with pIRES-*PROX1* or empty pIRES plasmid. Representative light microscope images were taken at a 200x magnification.

In order to determine whether Prox1 may affect the ability of tumor cells to invade through extracellular matrix, we measured cell migration in trans-well migration, wound-healing and Boyden chamber Matrigel invasion assays in FTC-133 depleted of Prox1 or overexpressing the protein. In four independent trans-well assays, we found that silencing *PROX1* significantly reduced migratory and invasion capacities (by over 65% and 75%, respectively) of si*PROX1*-FTC-133 cells in the presence of ECM proteins (Matrigel) compared to siNeg-FTC-133 cells (*P*-value < 0.0001; Figure 5A and 5B). This was in accordance with the wound healing assay results which confirmed that down-regulation of Prox1 expression was associated with a decreased motility of si*PROX1*-FTC-133 cells. Within 12 hours, the cells transfected with control negative siRNA reduced the wound surface by 85%, whereas FTC-133 cells transfected with si*PROX1* by merely 23% (Figure 5C), which represents an average four-fold reduction in the wound closure efficiency, as estimated based on three independent experiments. In contrast, overexpression of *PROX1* resulted in an enhanced cells motility (on average two-fold, *P*-value < 0.001) and Matrigel invasion (on average four times; *P*-value < 0.0001) as compared to control cells (Figure 6A and 6B). In wound healing assay, the scratch closure was on average four times faster in FTC-133 overexpressing Prox1 than in controls (Figure 6C).

**Figure 7:**
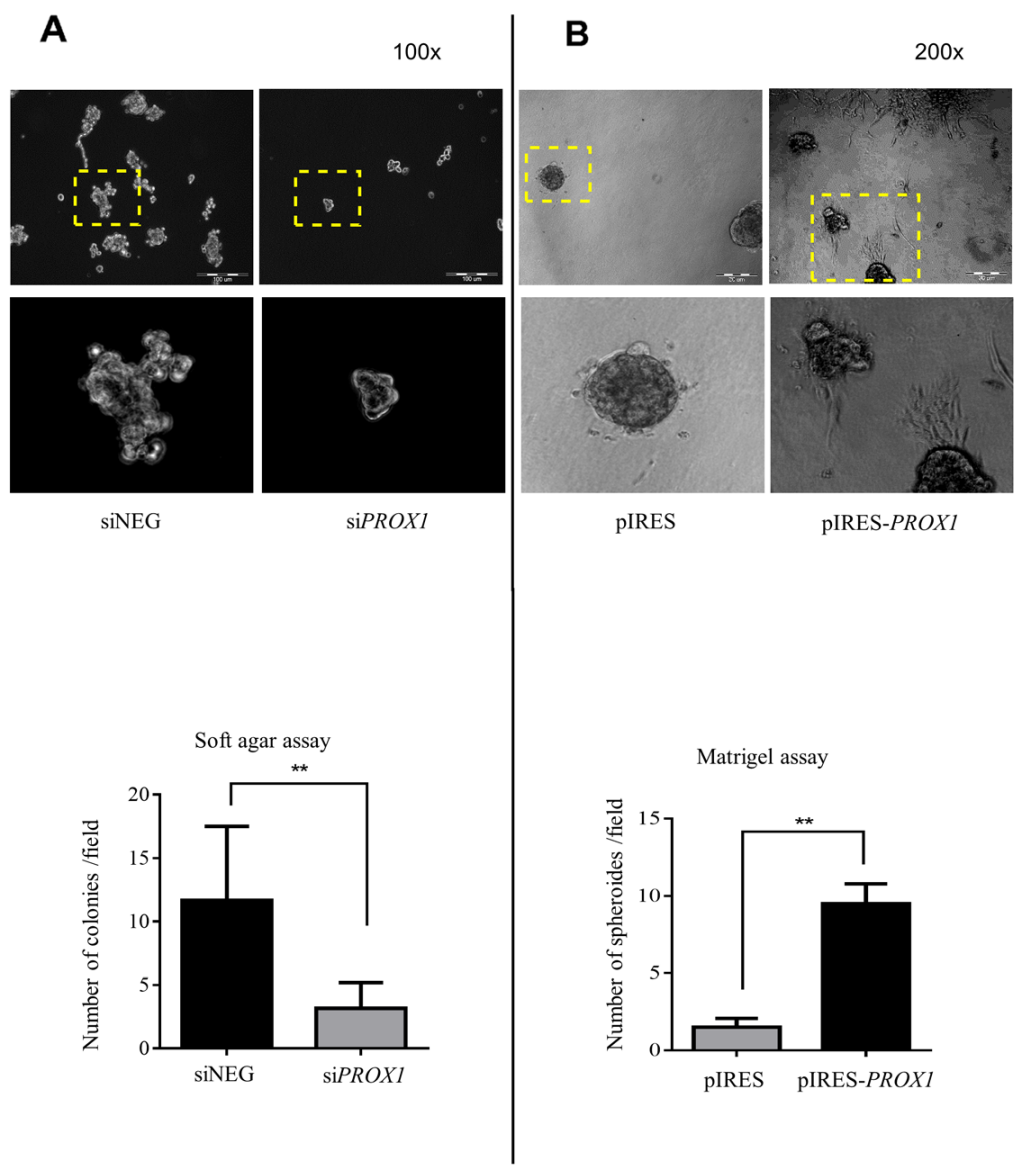
The effect of the *PROX1* knockdown **(A)** and overexpression **(B)** in FTC-133 cells on soft agar colony formation. After a transient transfection with si*PROX1* or pIRES-*PROX1*, the cells were seeded in 6-well culture plates containing soft agar. After two weeks, the colonies were stained and counted under a light microscope at a 100x or 200x magnification. The images show results representative of at least three independent experiments. The areas depicted by yellow boxes are zoomed in the lower panel. Lower panels show quantitative data for soft agar colonies and Matrigel spheroids formed in the two relevant assays. The *PROX1* silencing significantly reduced the rate of colony formation, whereas *PROX1* overexpression significantly promoted the rate of spheroid formation in FTC-133 cells. Bar graphs illustrate the means ± standard deviation (SD) from five assays, and asterisks (^**^) indicate the *P*-value < 0.01.

We also analyzed the effect of *PROX1* expression on the colony forming capacity of the FTC-133 cells using the Matrigel anchorage-independent growth assay which measures the ability of cells to proliferate in semi-solid conditions. The FTC-133/ si*PROX1* cells formed less colonies in soft agar than FTC-133/siNEG cells, and these colonies were smaller (Figure 7A). In contrast, FTC-133 cells overexpressing the Prox1 protein (FTC-133/pIRES-*PROX1* cells) formed a significantly higher number of colonies than the cells transfected with an empty plasmid. Moreover, they formed colonies in the form of large spheroids with the formation of cytoplasmic protrusion which extended into the Matrigel, whereas FTC-133/pIRES cells formed smaller and more regular flat colonies (Figure 7B).

**Figure 8:**
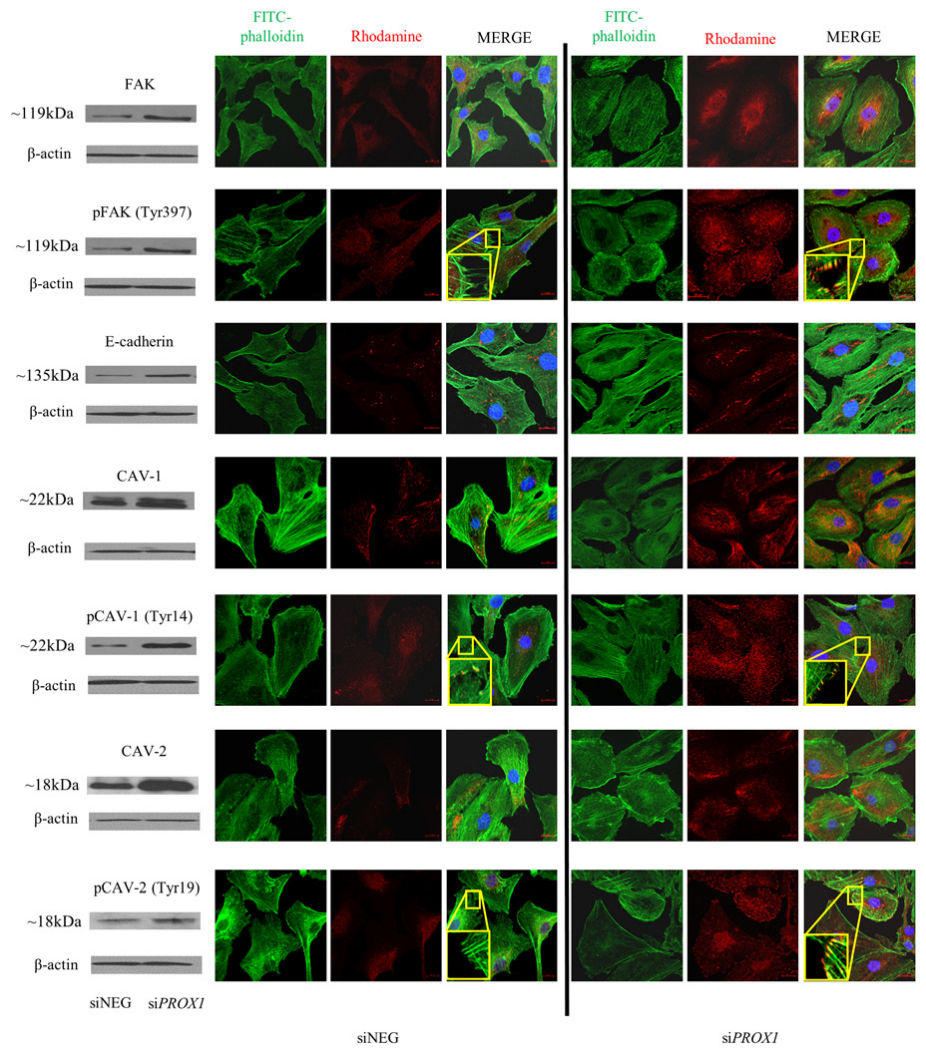
Immunofluorescent staining of FAK/pFAK CAV-1/pCAV-1, CAV-2/pCAV-2 and E-cadherin in FTC-133 cells depleted of Prox1 Fixed and permeabilized FTC-133 cells depleted of Prox1 or transfected with siNEG RNA were incubated overnight at 4°C with appropriate primary antibodies, followed by a one-hour room-temperature incubation with the appropriate secondary Rhodamine (TRICT)-conjugated AffiniPure F(ab’)_2_ fragment goat anti-rabbit IgG and DyLight549-conjugated AffiniPure goat anti-mouse IgG antibodies (red). Images are representative confocal micrographs of FTC-133/si*PROX1* cells in which F-actin was stained with phalloidin-AlexaFluor488 (green) and the nuclei (DNA) with DAPI (blue), taken at a 630x magnification. The yellow boxes depict the zoomed regions in the corresponding images.

In order to determine whether altered migration and colony formation capacities of the FTC-133 cells deprived of the Prox1 expression are associated with changes in the expression of proteins related to cell adhesion, we have analyzed by Western blot the expression of the following proteins involved in regulating cell adhesion: Focal Adhesion Kinase (FAK), E-cadherin, caveolin 1 (CAV-1) and caveolin 2 (CAV-2), both total (FAK, CAV-1, CAV-2) and phosphorylated (pFAK, pCAV-1 and pCAV-2) forms. We found that the levels of both total and phosphorylated FAK, CAV-1, and CAV-2 proteins were significantly higher in cells transfected with *PROX1* siRNA in comparison to the cells transfected with control siRNA, and these results were confirmed by immunofluorescent staining. An immunofluorescent analysis of their cellular localization has shown that the total FAK protein, expressed at low levels, localized around the nuclei in FTC-133/siNEG, whereas the expression of its phosphorylated form, pFAK was barely detected in the cytoplasm and not detected at all in the filopodia of these cells. In FTC-133/si*PROX1* cells however, pFAK was abundantly expressed in the cytoplasm, in particular around the nuclei, as well as at the numerous filopodia and focal contacts. Moreover, we observed an enhanced cytoplasmic and partly nuclear expression of total CAV-1 protein and CAV-2 protein in these cells, whereas their phosphorylated forms outside cytoplasm localized along actin stress fibers at the numerous elongated focal adhesions and filopodia. E-cadherin expression was also higher, however in both control and Prox1-deprived FTC-133 cells it was present only in the cytoplasm (Figure 8).

**Figure 9:**
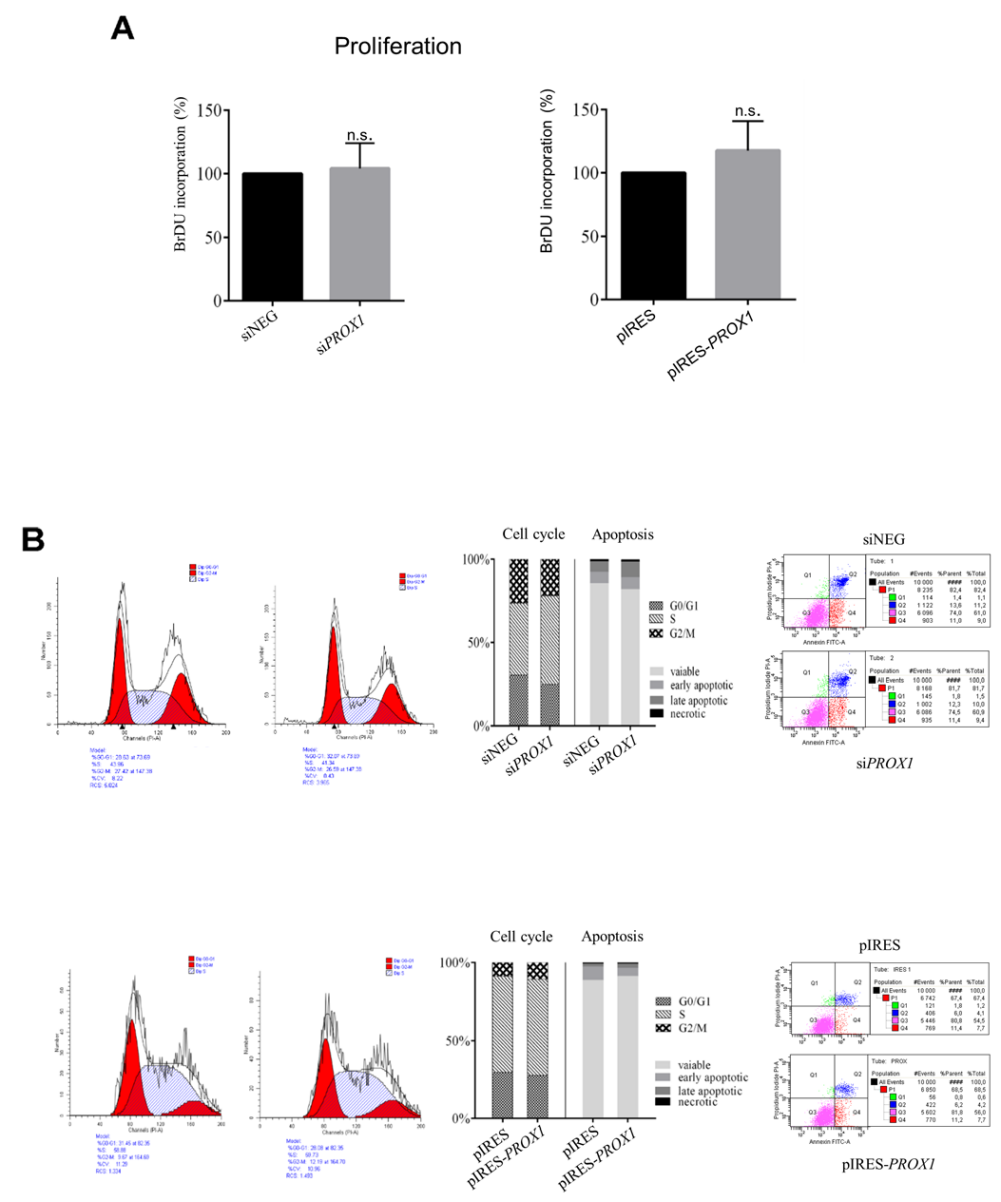
The effect of *PROX1* knockdown **(A)** and overexpression **(B)** on proliferation, cell cycle arrest, and apoptosis in FTC-133 cells. The cell proliferation was measured by the BrdU incorporation assay. Cell cycle profile was evaluated by propidium iodide staining and flow cytometry. Apoptosis rate was measured by propidium iodide and Annexin V-FITC staining followed by flow cytometry. Annexin V-FITC staining discriminates cells in early (lower right quadrant) and advanced (upper right quadrant) apoptotic states. Viable cells are double negative (lower left quadrant).

*PROX1* silencing and overexpression did not seem to affect cell proliferation. We detected no significant differences in apoptotic rate and the cell cycle characteristics between si*PROX1-* and siNEG-treated FTC-133 cells or between the FTC-133/pIRES-*PROX1* and the FTC-33/pIRES cells. Also the levels of DNA synthesis, as measured by the BrdU incorporation assay, remained unchanged despite altered Prox1 expression. Some changes in the viability of cells overexpressing Prox1 were detected. However, the results from five independent experiments did not reach statistical significance (Figure 9). Similar results were obtained with the plate colony formation assay (data not shown).

## DISCUSSION

The prospero-related homeobox 1 transcription factor (Prox1) is an evolutionarily conserved multifunctional protein which is well known for its essential physiological functions during the embryonic development of various organs and during lymphatic vasculogenesis [9, 12, 14, 17, 43, 44]. However, its involvement in the development of human cancers seems to be more complex and has not yet been fully elucidated. Dysregulation of *PROX1* expression has been detected in a variety of human cancers, which suggests that it may be involved in tumorigenesis [43]. Several studies have shown that *PROX1* may play different roles in different types of cancer, functioning as an oncogene in some of them and as a tumor suppressor gene in some others, depending on the type of cells that undergo transformation into cancer cells. Pro-oncogenic activity of *PROX1* was shown in colon cancer, renal carcinoma, and malignant astrocytic gliomas, whereas in pancreatic cancer, Kaposi sarcoma and kaposiform hemangioendothelioma, oral cancer, esophageal cancer, sporadic breast cancer, and neuroblastoma it was identified as a candidate tumor suppressor gene [24, 26, 27, 30, 35-37, 45-49]. There have been conflicting reports concerning the role of Prox1 in hepatocellular carcinoma, with some studies showing its oncogenic activity and some others suggesting it has tumor suppressive functions [28, 32, 50]. In papillary thyroid carcinoma, *PROX1* inactivation has been shown to promote malignant tumor behavior [39]. However, it is unclear whether and how the *PROX1* gene is involved in thyroid follicular cancer (FTC) tumorigenesis.

In this study, we show for the first time that Prox1 is specifically expressed at both transcript and protein level in cell lines derived from follicular thyroid cancer patients: FTC-133 and CGHT, as well as in archived FTC tissues (data not shown), with the Prox1 protein mainly localized in cell nuclei. In contrast, in thyroid cancer cell lines originating from papillary thyroid carcinoma (BcPAP and TPC1), only low levels of Prox1 mRNA were detectable while the corresponding protein was detected both by Western blot and immunofluorescent staining, even though at lower level than in the two FTC-derived cell lines. Of note, in PTC tissues, Prox1 expression was variable and localized mainly in the cytoplasm (data not shown). The normal thyroid follicular cells (NTHY) used as a control showed very low *PROX1* expression, at the transcript. However, a faint band corresponding to the Prox1 protein was also observed in the NTHY cells.

We found that *PROX1* expression is mediated by the phosphatidylinositol 3-kinase / serine-threonine protein kinase AKT (PI3K/AKT) signal transduction cascade. This signaling pathway plays an important role in many physiological processes, such as cell growth, proliferation and survival, and it is hyperactive in many cancers, including follicular thyroid carcinomas [51-53]. FTC-133 cells carry functionally inactivated *PTEN*, which results in a hyperactivation of the PI3K/AKT signaling pathway. In our study, the depletion of Prox1 did not seem to modify the PI3K/AKT pathway activity since the levels of both phosphorylated and total AKT protein did not change (Figure 2C). However, inactivating PI3K/AKT by a pharmacological inhibitor, LY294002, resulted in a significant decrease in the phosphorylated AKT form and Prox1 protein expression levels with no effect on the total AKT protein level. AKT is a downstream effector of PI3K. The inhibition of AKT1 and 2 kinases significantly reduced the phospho-AKT protein levels, whereas changes in the Prox1 protein expression were less manifested. Our results suggest that hyperactivation of the PI3K/AKT signaling pathway resulting from *PTEN* inactivation due to an inactivating mutation may regulate Prox1 expression in follicular carcinoma-originating FTC-133 cells. There is also a possibility that some alternative mechanisms for a constitutive induction of the PI3K/AKT pathway in cancer exist. Prox1 depletion induced a decrease in motility, migration and invasive potential of FTC-133 cells. This may have resulted from the deregulation of the PI3K/Akt signaling pathway independent of *PTEN* inactivation. Besides the PTEN inactivation-dependent activation of the PI3K/AKT pathway, also the Ras plays an important role in the activation of the PI3K/AKT pathway via interaction with its p110 catalytic subunit containing the Ras binding site. Furthermore, it can be assumed that genetic changes, such as mutations or the PI3KCA gene amplification, may be responsible for the activation of the PI3K/AKT pathway even though no genetic changes of PI3CA other that the PTEN inactivation have been reported for the FTC-133 cells. Moreover, AKT isoforms might play dual role in tumorigenesis, not only promoting tumorigenesis, but also suppressing migration and invasion, thus acting anti-oncogenically.

Of note, we did not observe changes in the expression of proteins from the ERK1/2 pathway in response to depletion or overexpression of Prox1, which suggests that *PROX1* is not involved in the regulation of this pathway in follicular thyroid cancer (data not shown).

It is well established that down-regulation of *PROX1* reduces the proliferation of colorectal, cervical cancer, oral, esophageal cancer cells and lung squamous-cell carcinoma cells whereas it significantly promotes the growth of hepatocellular carcinoma cells *in vitro* and does not significantly influence the proliferation rate of EOMA and Py-4-1 cells or renal cancer cells [28, 30-32, 45, 46, 48, 49, 54-56]. It was previously reported that restituting the Prox1 expression in a cell line originating from papillary thyroid carcinoma, BcPAP, reduces cell proliferation [39]. Our results for follicular thyroid cancer cells, FTC-133, show that modifying Prox1 expression does not influence follicular cell cycle, apoptosis or proliferation. This suggests that Prox1 activity is rather not dependent on the genetic background determining the regulation of these processes in follicular thyroid cancer cells. In the follicular cancer cells, Prox1 controls cytoskeleton arrangement, cell shape and polarity as well as focal adhesion. Cell senescence was not affected by modification of the Prox1 expression: we did not detect any changes in the expression levels of two senescence marker genes: GBL-1 and p16, by Q-RT-PCR and X-Gal cell staining (data not shown).

However, through loss-of-function and gain-of-function experiments we have shown that Prox1 expression is associated with the cell migration rate, invasive potential and anchorage-independent growth in FTC-133 cells. We observed that depleting *PROX1* reduced migration, motility, invasive potential, and a capacity of anchorage-independent growth of these follicular thyroid cancer cells *in vitro*, while Prox1 overexpression in the same cell line had a contrary effect. Moreover, the cells overexpressing Prox1 were more aggressive and mesenchymal in shape. All these results imply that Prox1 greatly promotes cell migration, motility and invasive potential *in vitro*, which suggests its involvement in the progression and spreading of follicular thyroid carcinoma cells.

The association between the Prox1 expression and invasive phenotype of the FTC-133 cells was further supported by the occurrence of changes in the cell cytoskeleton, adhesive properties and shape. The cytoskeleton is the cell structural support and it is essential for determining the cell shape. Its rearrangement is generally associated with alterations in cell migration and invasion capacities, and cell shape [57]. We found that cells with silenced *PROX1* acquired a rounder and flatter, amoeboid-like shape. This suggests that one of the primary responses observed in FTC-133 cells after loss of *PROX1* is the regulation of actin cytoskeleton, which in turn implies that Prox1 is involved in the modelling of actin cytoskeleton and in cell morphogenesis. Cells overexpressing *PROX1* became elongated, highly polarized, of a mesenchymal-like shape. Moreover, F-actin cytoskeleton changes were seen in both types of transfected cells, with the stress fibers redistributed around the periphery of cells.

Actin stress fibers are linked to the inner surface of plasma membrane through focal adhesion complex [58]. Migrating cells require both the formation and disassembly of focal adhesions which are under control of independent signaling pathways [59]. One of the key signaling molecules regulating focal adhesion turnover is focal adhesion kinase 2 (FAK2), a non-receptor tyrosine kinase which is involved in regulating many cellular processes, including adhesion, reorganization of the actin cytoskeleton, formation and disassembly of focal adhesions and cell protrusions, and motility signaling [60]. Prox1 was shown to mediate alterations of cell cytoskeleton, adhesiveness and cell shape as well as to induce cell migration in colon cancer cell lines [27]. In our study, the knock-down of *PROX1* induced overexpression of both total and phosphorylated FAK protein which localized centrally in the cytoplasm, mainly around cell nuclei. Phosphorylation of FAK is a post-translational modification involved in the cell adhesion signaling, regulating the cell focal adhesion capacities, and our results suggest that Prox1 contributes to the regulation of cell adhesion by regulating FAK phosphorylation.

Adhesion dynamics is also controlled by the membrane domain organization. Its key structural constituents: small invaginated plasma membrane domain caveolae, in particular Caveolin-1 (Cav-1) and -2, are frequently associated with actin cytoskeleton through stress fibers. FTC-133 cells with suppressed *PROX1* expression showed elevated expression levels of both Cav-1 and Cav-2, which localized mainly in the intracellular compartment of cells, in the perinuclear area. It is worth noting that expression of both Cav proteins in FTC had been shown to be decreased in thyroid carcinoma [61]. It has been hypothesized that Cav-1 may participate in focal adhesion turnover, with some studies suggesting that it may act either as a tumor suppressor, or as a tumor-promoting factor, depending on the cellular context [62-64]. The role of Cav-2 in the focal adhesion dynamics though remains poorly known.

We also found that active phosphorylated Cav1-Tyr14 and Cav2-Tyr19 variants were highly expressed and mainly localized to the abundant focal adhesions within the cells and on the plasma membranes of follicular cancer cells, towards the endings of stress fibers, when the focal adhesions formed. These results clearly demonstrate that *PROX1* depletion in follicular cancer cells promotes phosphorylation of Cav-1 and Cav-2 and increases the formation of focal adhesions with the participation of phosphorylated Cav-1 and -2. Another molecule which is very important for cell-cell adhesion is E-cadherin which forms complexes with β-catenin [65]. Therefore, we analyzed the expression levels of E-cadherin and its cellular distribution in the follicular cancer cells with the knocked-down *PROX1* gene. The E-cadherin levels were higher in these cells than in control cells, however in both types of cells E-cadherin localized cytoplasmically and was exclusively distributed along the stress fibers. We observed a significant reduction of the migratory and invasive potential in FTC-133 cells depleted of *PROX1*. In contrast, FTC-133 cells overexpressing *PROX1* had an enhanced migratory and in particular invasive potential. The altered cell migration capacities and invasiveness could be attributed to the changed proliferative potential of transfected cells. However, it should be noted that the *in vitro* proliferative activity and viability of the FTC-133 cells did not substantially change following modifications of the *PROX1* gene expression. As mesenchymal cells show a higher migratory and invasive potential, malignant cancer cell phenotype is directly dependent on epithelial to mesenchymal transition [66]. The induction of cell migration comprises, among others, changes in the organization and distribution of F-actin filaments and the formation of membrane protrusions. Indeed, the FTC-133 cells deprived of Prox1 developed strong actin stress fibers and numerous pseudopodia, filopodia and lamelipodia, suggesting that Prox1 expression may influence the migratory potential of the cells by regulating the cytoskeleton rearrangement.

In conclusion, our data strongly support a hypothesis that *PROX1* may play an important role in tumorigenesis and progression of follicular thyroid carcinoma. Given that Prox1 expression and function depend on the tissue and cell context, further studies should focus on identifying genes whose products regulate *PROX1* expression in follicular thyroid cancer in order to elucidate the precise function of this protein in FTC development and progression.

## MATERIALS AND METHODS

### Cell lines and cell culture

Four cell lines derived from human thyroid carcinoma were used in this study: two originating from the papillary type (TPC1 and BcPAP) and two originating from the follicular type (FTC-133 and CGTH-W-1). BcPAP and CGTH-W-1 cell lines were purchased from the German Collection of Microorganisms and Cell Cultures (DSMZ, Braunschweig, Germany, ref. ACC-272 and ACC-360 respectively). The FTC-133 cell line was obtained from the European Collection of Cell Cultures (ECACC), UK, 94060901 and the TPC1 cell line was kindly provided by Dr. M. Santoro, University Federico II, Naples, Italy. Normal human thyroid epithelial SV40T-immortalized cells (Nthy-ori 3-1) obtained from ECACC, UK (ref. 90011609) and a human hepatocellular carcinoma cell line (HepG2) purchased from the American Type Culture Collection (ATCC, USA, ref. HB-8065), in which high *PROX1* expression had been described [40] were used as controls. The cell lines were cultured as previously described [41]. In brief, TPC1, BcPAP, CGTH-W-1 and NTHY cells were cultured in Roswell Park Memorial Institute medium 1640 (RPMI-1640) supplemented with 10% (v/v) heat-inactivated fetal bovine serum (FBS; Gibco, Invitrogene, Carlsbad, CA, USA). The FTC-133 and HepG2 cell lines were maintained in Dulbecco’s modified Eagles’s medium (DMEM) : F-12 (1:1), supplemented with 10% fetal bovine serum (Gibco, Grand Island, NY, USA). All cell lines were grown at 37°C in humidified 5% CO_2_/95% air atmosphere. The cells were put in culture from frozen stocks two passages before the experiments andmaintained in culture for a maximum of 10 passages. The number of viable cells was estimated by Trypane Blue exclusion with automatic counting on EVA Automatic Cell counter (Nano EnTek, Korea). All cell lines used in this study were regularly tested for the presence of mycoplasma contamination using a PCR-based method as described elsewhere [42].

### RNA extraction and quantitative real-time PCR (Q-RT-PCR)

Total RNA was extracted from cultured cells using GeneMATRIX Universal Purification Kit (EURx, Gdansk, Poland) followed by treatment with on-column DNAse (A&A Biotechnology, Gdynia, Poland) according to the manufacturers’ protocols. RNA quality and concentration were evaluated using a Synergy 2 Multi-Mode Reader (BioTek Instruments, Winooski, VT, USA). Only samples with the A260/A280 ratio close to 2.0 were used for further analyses.

To determine the *PROX1* expression in the cell lines, 1μg of total RNA was reverse-transcribed to cDNA using a High Capacity cDNA Reverse Transcription Kit (Applied Biosystems, Foster City, Ca, USA). Transcript levels of human *PROX1* gene and the *ACTB* reference gene were quantified by RT-qPCR using Maxima™ SYBR Green/Fluorescein qPCR Master Mix (Life Technologies, Carlsbad, Ca, USA) and specific oligonucleotide primers: *PROX1* (NM_001270616) Forward: 5’-CCAGCTCCAATATGCTGAAGACCTA-3’, Reverse: 5’-CATCGTTGATGGCTTGACGTG-3‘, amplicon size 144 bp; *ACTB* (NM_001101) Forward: 5’-FGCCGAGGACTTTGATTGC-3’; Reverse: 5’-CTGTGTGGACTTGGGAGAG-3’ (amplicon size: 146 bp).

Following the amplification (reverse transcriptase inactivation for 5 min at 95°C, then 40 cycles of denaturation at 95°C for 30 s, 15s of primers annealing at 58°C and 10s of primers extension at 72°C), melting curves were obtained by performing a temperature gradient from 65 to 90°C.

Amplification, data acquisition and data analysis were performed using the CFX Connect Real-Time PCR Detection System and software (Bio-Rad, Hercules, Ca, USA). All assays were run in triplicates in each of at least three independent biological experiments. Primer reaction specificity was confirmed by both agarose gel electrophoresis and melting curve analysis. C_t_ values for the *PROX*1gene were normalized against the endogenous *ACTB*control gene using the ΔΔCt method and the relative gene expression was analyzed using the CFX Manager software (Bio-Rad).

### Determining the Prox1 protein levels by western blot

Preparation of total protein extracts from whole cells and Western blotting were carried out as previously described using the following antibodies: goat antibody against human Pro2-Gln259 Prox1 (AF2727, R&D Systems, Minneapolis, MN USA) diluted 1:2000 or rabbit anti-human against Prox1 (Cell Signaling #13910) diluted 1:1000 as primary antibodies, followed by a HRP-conjugated secondary antibody: a rabbit anti-goat secondary antibody (Jackson ImmunoResearch Laboratories, Vest Grow, Pa, USA) diluted 1:20,000 or a goat anti-rabbit antibody (DAKO, Carpinteria, CA, USA) diluted 1:5000, depending on the origin of the primary antibody [41]. The primary antibodies had been previously validated for use in Western blot, immunofluorescent and immunohistochemical analyses as the most reliable out of four different antibodies tested to specifically detect the Prox1 protein. The proteins were then detected using SuperSignal West Dura Chemiluminescent Substrate (Pierce Biotechnology, Rockford, IL, USA) according to standard procedures. The membranes were re-probed with a monoclonal mouse anti-β-actin antibody (diluted 1:5000; Sigma-Aldrich, USA) to verify equal protein loading.

### Cell fractionation

All the following steps were performed on ice or at 4° C. Cells were washed with cold PBS, centrifuged at 1000 rpm for 5 minutes, re-suspended in ice-cold buffer A (10 mM HEPES, pH 7.9, 1.5 mM MgCl_2_, 0.5 mM dithiotreitol, 1x complete protease and phosphatase Inhibitor cocktail (Roche, Basel, Switzerland), and 0.5 mM PMSF), homogenized with a loose-fitting Dounce homogenizer (30 strokes), and incubated on ice for 30 min. The supernatant containing the cytosolic fraction was collected following a centrifugation of the homogenate at 1,200 rpm for 5 minutes and stored at -80°C until use. The pellet was washed twice with buffer A and then re-homogenized (30 strokes) in buffer B (20 mM HEPES pH7.9, 420 mM NaCL, 1.5 mM MgCl_2_, 25 % glycerol, 0.5 mM DTT, 1x of complete protease and phosphatase inhibitor cocktail (Roche) and 0.5 mM PMSF), incubated on ice for 30 minutes, and centrifuged at 13,000 rpm for 5 minutes. The supernatant (nuclear fraction) was then collected and stored at-80°C until use. The purity of the fractions was validated with antibodies detecting HSP-90 (cytosolic) and PARP (nuclear), (ProteoExtract S-PEK Antibody Control Kit, Millipore, Billerica, Ma, USA).

### Silencing *PROX1* by short interfering RNA (siRNA)

FTC-133 cells were used to study the effect of *PROX1* knock-down on hallmark characteristics of malignant cells: migration, invasion, motility, proliferation, apoptosis and anchorage–independent growth. Cells were grown to 60-70% confluence in 24-well or 6-well plates and transiently transfected with either non-targeted pool of small interfering RNAs (MISSION^®^ siRNA Universal Negative Control #1, Sigma-Aldrich, St. Louis, MO, USA), a pool of specific siRNAs against human *PROX1* (MISSION^®^ esiRNA, Sigma-Aldrich, EHU053851) or with *PROX1* siRNA (h) (sc-106451, Santa Cruz Biotechnology, USA), using Lipofectamine^®^ 2000 Reagent (Life Technologies, Carlsbad, CA, USA) according to the manufacturers’ protocol. The optimal transfection efficiency was obtained using a cell density of 4.5x10^3^ per well, 30 nM siRNAs in OptiMem medium (Roche, Basel, Switzerland), and 1.5 μl Lipofectamine/OptiMem. Cells were incubated with the transfection mix for 48 hours and then harvested for RNA purification or used for other assays. The experiments were conducted on at least three different cell passages and run in triplicate (three samples of the same cell passage).

### Prox1 overexpression by transient transfection with pIRES2-eYFP-*PROX1* plasmid

FTC-133 cells were grown to 60-70% confluence, washed with cold phosphate buffered saline and tripsinized, centrifuged, and then re-suspended in DMEM/F-12 medium with 10% FBS. Approximately 3x10^5^ cells were transiently transfected with 1 μg of YFP-expressing plasmid pIRES2-eYFP-PROX1 (pIRES-PROX1) or empty plasmid pIRES2-eYFP (pIRES; both plasmids kindly provided by Dr. J. Becker, Department of Anatomy and Cell Biology, Universitätsmedizin Göttingen UMG, Göttingen, Germany) using LIpofectamine 2000 (Invitrogen, Carlsbad, CA, USA) according to the manufacturer’s instructions. Cells were harvested at 48 hours following transfection, the transfection efficiency was verified under an inverted fluorescence microscope (AxioObserver D1, Zeiss, Germany), and the cells were used in functional assays. The experiments were conducted on at least three different cell passages and run in triplicate (three samples of the same cell passage).

### Analysis of the association between *PROX1* expression and the PI3K/Akt pathway

In order to test whether there is a relationship between Prox1 expression and the genetic alterations in the PI3K/Akt signaling cascade, we first assessed the impact of *PROX1* knock-down on the expression of AKT and pAKT proteins, and then we sequentially inhibited two elements of this pathway: PI3K with the LY294002 inhibitor and Akt1/Akt2 with A6730. To this effect, FTC-133 cells were grown in standard conditions up to 70% of confluence. Then, the medium was replaced with culture medium containing either a PI3K inhibitor, LY294002 (50 μM), or an AKT1/2 inhibitor, A6730 (10 μM), dissolved in DMSO (Sigma Chemical, St. Louis, MO), and the cells were incubated for 24 hours. After that time, the media was changed and the cells were incubated in standard culture media for another 24 hours. Cell cultures in which media with DMSO only was added were used as a control. The concentration of DMSO in both control and test cultures did not exceed 0.5%. Afterwards, whole cell protein extracts were prepared and analyzed by Western blot as described in this report for Prox1 expression analysis. The antibodies used are listed in Table 1. All secondary antibodies were conjugated with horseradish peroxidase (HRP). Antibody binding was then visualized as described elsewhere [41].

**Table 1:** Primary and secondary antibodies used to analyse the expression of Prox1 and other proteins by western blotting and immunochistochemistry

Analyzed protein	Primary antibody-Western blotting dilution	Secondary antibody - Western blotting dilution
Prox1	Goat anti-human Prox1 (1:2000), R&D System, USA	Affinity-purified rabbit anti-goat antibody/HRP (1:20000), Jackson ImmunoResearch Laboratories, USA
Akt	Rabbit anti-phospho AKT (Ser473) (1:1000) or rabbit pan-AKT (1:1000), both from Cell Signaling Technology, USA	Polyclonal goat anti-rabbit immunoglobulins/HRP (1:5000), DAKO, Denmark
Hsp90	Mouse monoclonal anti-Hsp90 (EMD-17D7) (1:5000), EDM Millipore, USA	Affinity-purified goat anti-mouse antibody (1:10000), Jackson ImmunoResearch
PARP1	Mouse monoclonal anti-PARP1(Ab-2) (C-2-10) (1:5000), EDM Millipore, USA	Affinity-purified goat anti-mouse antibody (1:10000), Jackson ImmunoResearch
FAK	Rabbit polyclonal anti-human FAK (1:1000), Cell Signaling Technology, USA	Polyclonal goat anti-rabbit immunoglobulins/HRP, DAKO, Denmark (1:5000)
pFAK (Tyr397)	Rabbit polyclonal anti-phospho FAK (Tyr397) (1:1000), Cell Signaling Technology, USA	Polyclonal goat anti-rabbit immunoglobulins/HRP, (1:5000) DAKO
E-Cadherin	Purified mouse anti-E-cadherin Clone 34/E-cadherin (RUO), 1:5000, BD Biosciences, USA	Affinity-purified goat anti-mouse antibody (1:10000), Jackson ImmunoResearch
CAV-1	Rabbit mAb monoclonal anti-human Caveolin-1 (1:1000), Cell Signaling Technology, USA	Polyclonal goat anti-rabbit immunoglobulins/HRP, (1:5000) DAKO
pCAV-1 (Tyr14)	Rabbit polyclonal anti-phospho-Caveolin-1 (Tyr14) (1:1000) Cell Signaling Technology, USA	Polyclonal goat anti-rabbit immunoglobulins/HRP, (1:5000), DAKO
CAV-2	Rabbit polyclonal anti- human Caveolin-2 (1:500), Abcam, UK	Polyclonal goat anti-rabbit immunoglobulins/HRP, DAKO,
pCAV-2 (Tyr19)	Rabbit polyclonal anti- human Phospho-Caveolin-2 (Tyr19) (1:500), Abcam, UK	Polyclonal goat anti-rabbit immunoglobulins/HRP, (1:5000), DAKO
β-actin (loading control)	Mouse monoclonal anti-β-actin (1:5000), Sigma –Aldrich, USA	Affinity-purified goat anti-mouse antibody (1:10000), Jackson ImmunoResearch
**Immunofluorescence analysis**		
	**Primary antibody- IF dilution**	**Secondary antibody – IF dilution**
Prox1	Goat anti-human Prox1 (1:50), R&D System, USA	DyLight® 550-affinity purified rabbit F(ab’)2 anti-goat IgG - H&L, (1:500), Abcam
FAK	Rabbit polyclonal anti-human FAK (1:500), Cell Signaling Technology, USA	Rhodamine (TRITC) AffinityPure goat anti-rabbit IgG (H+L), (1:100), Jackson ImmunoResearch Laboratories, USA
FAK	Rabbit polyclonal anti-human FAK (1:500), Cell Signaling Technology, USA	Rhodamine (TRITC) AffinityPure goat anti-rabbit IgG (H+L), (1:100), Jackson ImmunoResearch Laboratories, USA)
pFAK (Tyr397)	Rabbit polyclonal anti-phospho FAK (Tyr397) (1:500), Cell Signaling Technology, USA	Rhodamine (TRITC) AffinityPure goat anti-rabbit IgG (H+L), (1:100), Jackson ImmunoResearch Laboratories, USA)
E-Cadherin	Purified monoclonal Mouse anti-E-Cadherin Clone 34/E Cadherin (RUO), 1:5000, BD Biosciences, USA	Rhodamine (TRITC) AffinityPure goat anti-rabbit IgG (H+L), (1:100), Jackson ImmunoResearch Laboratories, USA)
CAV-1	Rabbit mAb monoclonal anti-human Caveolin-1 (1:500), Cell Signaling Technology, USA	Rhodamine (TRITC) AffinityPure goat anti-rabbit IgG (H+L), (1:100), Jackson ImmunoResearch Laboratories, USA)
pCAV-1 (Tyr14)	Rabbit polyclonal anti-phospho-Caveolin-1 (Tyr14) (1:500), Cell Signaling Technology, USA	Rhodamine (TRITC) AffinityPure goat anti-rabbit IgG (H+L), (1:100), Jackson ImmunoResearch Laboratories, USA)
CAV-2	Rabbit polyclonal anti- human Caveolin-2 (1:500), Abcam, UK	Rhodamine (TRITC) AffinityPure goat anti-rabbit IgG (H+L), (1:100), Jackson ImmunoResearch Laboratories, USA)
pCAV-2 (Tyr19)	Rabbit polyclonal anti- human Phospho-Caveolin-2 (Tyr19) (1:500), Abcam, UK	Rhodamine (TRITC) AffinityPure goat anti-rabbit IgG (H+L), (1:100), Jackson ImmunoResearch Laboratories, USA)

### *In vitro* cell proliferation assay

Bromodeoxyuridine (BrdU) incorporation assay (Millipore, Billerica, MA, USA) was used according to the manufacturer’s instructions to assess cell proliferation by quantifying DNA synthesis. In brief, 8x10^3^ cells per well were seeded in 96-well plates and the *PROX1* gene knockdown or overexpression were performed. After 48 hours, the cells were labeled with BrdU by incubation for 17 hours at 37°C. Next, they were fixed and incubated with the peroxidase-conjugated anti-BrdU antibody for one hour. The immune complexes of the anti-BrdU antibody bound to BrdU incorporated into newly synthesized cellular DNA were then detected by a reaction with the 3, 3’, 5 5’-tetramethylbrenzidine (TMB) substrate. After a 30-minute incubation, the reaction was stopped by adding a blocking solution (2.5 N H_2_SO_4_) and the absorbance was measured at 450 nm with a reference wavelength of 595 nm using the Synergy 2 Multi-Mode Reader (BioTek, Winooski, VT, USA). Cells incubated with standard culture medium were used as controls. Each sample was assayed in five replicates.

### Flow cytometric analysis of apoptosis

Detection of apoptotic FTC-133 cells was performed using the Annexin V-FITC Apoptosis Detection Kit (Abcam, UK) as previously described [41]. Briefly, 48 hours after siRNA or pIRES-PROX1 transfection, the cells were harvested, washed with PBS, and incubated with Annexin V-fluorescein isothicyanate (FITC) and propidium iodide for 5 minutes at room temperature. The stained cells were then analyzed by flow cytometry (Ex 488 nm, Em 530 nm, FACSCantoII, BD Biosciences, USA). The cells transfected with non-specific negative siRNA and non-transfected cells were used as controls. This analysis was performed in three independent biological experiments, each in triplicate.

### *In vitro* migration and invasion assay

For trans-well migration assay, the cell suspension containing 1x10^5^ cells per well was plated in an insert of the Boyden chamber with 8-μm pores containing non-coated membrane (BD Bioscience, San Jose, CA, USA), whereas for the analysis of the cell invasive potential, 1x10^5^ cells per well were plated in an insert containing Matrigel-coated membrane (BD BioCoat Tumor Invasion System, BD Bioscience, 8 μm pore size), in triplicates per condition. In both assays, cells were plated in serum-free medium and medium supplemented with 10% FBS was added to the lower chamber as a chemoattractant. After 24 hours of incubation, the cells that migrated to the bottom surface of the insert membrane were fixed and stained using the Diff-Quick Stain Set (Medion Diagnostics, Düdingen, Switzerland) staining the cytoplasm in red (eosin G) and nuclei in blue (thiazine dye). The membranes were then cut out and mounted on microscopic slides. Five fields of each insert were randomly chosen and photographed under a light microscope (Olympus BX41) at a 40x magnification, and the cells within these fields were counted. Each experiment was performed in triplicate and repeated at least three times. The data were summarized as means with standard deviation (SD) and presented as a percentages relative to controls.

### Wound healing assay

The migratory capacity of FTC-133 cells was determined by a wound healing assay as described elsewhere [41]. Briefly, the transfected cells were plated in 6-well culture plates and grown to a confluent cell monolayer. After scratching with a sterile 200 μl pipette tip, wells were washed with PBS to eliminate free-floating cells and debris. Then, the width of the scratch (wound) was measured. The rate of the wound closure was monitored for 24 hours at six-hour intervals under a microscope (AxioObserver D1, Zeiss Jena at 100x magnification). After 24 hours, the width of the remaining gap (scratch) was measured on phase-contrast pictures from at least five independent fields using the Image J software (National Institute of Health, Bethesda, MD). The relative degree of wound closure (relative Δwound area) was calculated as Δwound area of transfected cells as compared to that of negative controls (siNeg-FTC-133 or FTC-133/pIRES cells). The migration distance was calculated according to the following formula: percent of initial wound width at time zero minus percent wound width at 24h. All experiments were performed in triplicates and repeated at least three times.

### Anchorage-independent growth assay

The capacity of transfected cells for anchorage-independent growth was determined using a colony forming assay in soft agar (for FTC-133 cells in which *PROX1* expression had been silenced) or a Matrigel assay (for cells overexpressing Prox1). Two days after transfection, 5x10^3^ cells were suspended in DMEM/F-12/10% FBS/0.35% agarose (Roth, Germany) or in 2.5% Matrigel (Sigma-Aldrich, St. Louis, Missouri, USA) diluted with DMEM/F-12/10% FBS, and seeded in 6-well plates containing DMEM/F-12/10% FBS /0.76% agar (Carl Roth, Karlsruhe, Germany) or in 4-well Lab-Teck chamber slides (ThermoFisher Scientific, Waltham, Massachusetts USA) coated with 100% Matrigel, respectively. The cells were then incubated at 37°C in humidified 5%CO2/95% air atmosphere for two weeks, with the culture medium being changed every three days, until visible colonies formed. Afterwards, the plates were incubated with crystal violet in methanol to fix and visualize cell colonies. Their number and size were then evaluated and the colonies photographed under the AxioObserver D1 microscope (Zeiss, Essen, Germany) at a 100x and 400x magnification. All experiments were run in triplicates and repeated at least three times.

### Determining protein expression and localization by immunofluorescence

Cells in DMEM/F-12 /10% FBS medium were plated onto glass coverslips for 12 hours (reaching about 70% of confluency), and then fixed with 4% paraformaldehyde. Afterwards, they were permeabilized with 0.25% Triton X-100 in PBS, blocked with 3% BSA in PBS for one hour, and incubated overnight at 4° C with a specific primary antibodie (Table 1). Next, the cells were incubated with either the DyLight 594-affinity purified rabbit F(ab)_2_ anti-goat IgG or with the Rhodamine (TRICT) affinity pure goat anti-rabbit IgG (H+L) secondary antibody (red fluorescence; Table 1) for one hour at room temperature. To visualize filamentous F-actin, the cells were stained with FITC-labelled Phalloidyn (2μg/ml, 1:500, green fluorescence), according to the manufacturer’s protocol. The nuclei were stained with 4’, 6-diamidino-2-phenylindole (DAPI; 2μg/ml’ blue). An extensive wash was performed between all steps. The cover slips were then mounted onto slides and images were captured using the inverted fluorescence AxioObserver D1 Microscope (Zeiss, Germany) with a 1000x oil-immersion lens or a laser scanning confocal microscope (LSM800 equipped with ZEN2.1 software, AxioObserver Z1, Zeiss). Experiments were run in triplicates and repeated at least three times.

### Statistical analysis

All experiments were performed in triplicates and repeated at least three times, unless stated otherwise. Statistical analyses were performed using GraphPad Prism (SPSS Inc., Chicago, IL, USA). *P*-values below 0.05 were considered as indicative of a statistical significance.

## Abbreviations

ACTB: beta-actin gene
AKT: protein kinase B
BrdU: bromodeoxyuridine
BSA: bovine serum albumin
CAV: caveolin
DAPI: 4’,6-Diamidino-2-Phenylindole
DMEM: Dulbecco’s modified Eagle’s medium
DTC: differentiated thyroid carcinoma
FAK: focal adhesion kinase
FBS: fetal bovine serum
FITC: fluorescein isothiocyanate
FTC: follicular thyroid carcinoma
YFP: yellow fluorescent protein
HRP: horseradish peroxidase
PI3K: phosphatidylinositol 3-kinase
PBS: phosphate buffered saline
PROX1: Prospero homeobox protein 1
PTC: papillary thyroid carcinoma
PTEN: phosphatase and tensin homolog
qPCR: quantitative polymerase chain reaction
RT-PCR: real-time polymerase chain reaction
SD: standard deviation
VEGFR: vascular endothelial growth factor receptor
